# Effectiveness of hand hygiene interventions in reducing illness absence among children in educational settings: a systematic review and meta-analysis

**DOI:** 10.1136/archdischild-2015-308875

**Published:** 2015-10-15

**Authors:** Micky Willmott, Alexandra Nicholson, Heide Busse, Georgina J MacArthur, Sara Brookes, Rona Campbell

**Affiliations:** School of Social and Community Medicine, University of Bristol, Bristol, UK

**Keywords:** Infectious Diseases, School Health, Respiratory

## Abstract

**Objective:**

To undertake a systematic review and meta-analysis to establish the effectiveness of handwashing in reducing absence and/or the spread of respiratory tract (RT) and/or gastrointestinal (GI) infection among school-aged children and/or staff in educational settings.

**Design:**

Randomised-controlled trials (RCTs).

**Setting:**

Schools and other settings with a formal educational component in any country.

**Patients:**

Children aged 3–11 years, and/or staff working with them.

**Intervention:**

Interventions with a hand hygiene component.

**Main outcome measures:**

Incidence of RT or GI infections or symptoms related to such infections; absenteeism; laboratory results of RT and/or GI infections.

**Results:**

Eighteen cluster RCTs were identified; 13 school-based, 5 in child day care facilities or preschools. Studies were heterogeneous and had significant quality issues including small numbers of clusters and participants and inadequate randomisation. Individual study results suggest interventions may reduce children's absence, RT infection incidence and symptoms, and laboratory confirmed influenza-like illness. Evidence of impact on GI infection or symptoms was equivocal.

**Conclusions:**

Studies are generally not well executed or reported. Despite updating existing systematic reviews and identifying new studies, evidence of the effect of hand hygiene interventions on infection incidence in educational settings is mostly equivocal but they may decrease RT infection among children. These results update and add to knowledge about this crucial public health issue in key settings with a vulnerable population. More robust, well reported cluster RCTs which learn from existing studies, are required.

**What is already known on this topic**

- As semiclosed settings where large numbers of children with immature immunity regularly congregate, educational establishments are potentially effective places to prevent spread of infection.
- Evidence is equivocal but potentially promising for the effectiveness of hand hygiene interventions in preventing the spread of respiratory tract and gastrointestinal infection.
- Three systematic reviews of studies of hand hygiene interventions to prevent respiratory and/or gastrointestinal infections focus on educational settings; each has significant limitations.

**What this study adds**

- Eighteen cluster randomised controlled trials of the effectiveness of hand hygiene interventions in educational settings were identified; more than in previous dated reviews.
- Study design and reporting standards are generally low quality, impeding meta-analyses, but recently published studies show signs of improvements.
- Evidence of the impact of hand hygiene interventions among this population remains equivocal: this review makes recommendations for improving future trials to evaluate interventions.

## Introduction

Young children are particularly susceptible to respiratory tract (RT) and gastrointestinal (GI) infections. While usually self-limiting, these highly infectious illnesses spread quickly in semiclosed settings such as schools. Infections affect child health, causing missed educational opportunities which may have a detrimental effect on educational outcomes,^1^ ^2^ lost productivity and days off work for school staff.^3^ Educational settings where large numbers of children with immature immunity congregate are promising sites for preventing infection, particularly as outbreaks can affect whole schools and spread to vulnerable populations (eg, younger siblings) in the community.^4^ ^5^

Several systematic reviews (SRs) have evaluated evidence of interventions to prevent RT and GI infections;^6–16^ current evidence is equivocal but promising for the effectiveness of hand hygiene interventions in preventing RT and GI infection. Four SRs have included studies evaluating interventions in educational settings alongside other settings;^8^ ^9^ ^11^ ^14^ two focus on RT infection,^11^ ^14^ two focus on diarrhoea prevention.^8^ ^9^ Two of these are Cochrane reviews;^8^ ^11^ one recommended that: “effort should be concentrated on reducing transmission from young children through regular education at school on hygiene” (ref.^11^, p.9).

Three SRs^12^ ^13^ ^16^ focus exclusively on studies among children in educational settings. However, one only included hand sanitiser interventions;^13^ another included children 2–11 years old and is over a decade old.^16^ The most recent SR focused on the effects of multicomponent interventions (access to safe water, handwashing facilities, hygiene education) but did not assess study quality, included numerous study designs and had limited search parameters (eg, only searched in two databases).^12^ None of these SRs included meta-analyses (MAs). This review aimed to update these reviews using thorough methods (eg, searching a range of databases) to identify all relevant studies which apply the most robust study design (randomised controlled trial, RCT) for evaluating interventions.

The objective of this SR was to summarise evidence of the effectiveness of hand hygiene interventions in reducing infectious illness and/or absence in educational settings for children aged 3–11 years and/or staff working with them, and to obtain a quantified estimate of the effect using MAs if possible.

## Methods

This SR is reported in line with current guidance.^17^ Review coauthors agreed the review protocol.^18^

### Eligibility criteria

This SR included RCTs of interventions with a hand hygiene component (any comparator) in educational settings for children aged 3–11 years in any country. No length of follow-up was defined.

Educational settings were defined as institutions incorporating formal educational activities including day care facilities and nurseries. Other community settings (eg, playschools) and domestic child care settings were excluded. Study populations could include staff and/or children in these settings. The review age range aimed to ensure the inclusion of all studies in formal educational settings for younger (primary or elementary school-aged) children—hereafter referred to as primary school-aged children—where children can be expected to understand hand hygiene, toilet themselves and clean their own hands. Study populations could include children whose age overlapped with the review age range (eg, 2–6-year-old, 5–12 year-old) because school policy and practice varies between countries: children start formal education at different ages; children may repeat a year so may be older than 11 years in primary school; structured nursery facilities for younger children may be integrated in schools.

Hand hygiene interventions were defined as any initiative for children and/or staff working with them undertaken to prevent the spread of infectious illness. Comparators could include placebos or active comparators such as handwashing with soap compared with hand sanitiser use.

Inclusion criteria were piloted on reports known to authors.

Primary review outcomes were: incidence of RT or GI infections or symptoms related to such infections; absenteeism rate; or laboratory results of RT and/or GI infections. Secondary outcomes were: hospital admissions due to such infections; changes in knowledge, attitudes, beliefs or behaviours about hand hygiene among children and/or staff working with them. We intended that outcomes related to children and staff be considered separately: we did not anticipate many studies would report staff outcomes. Studies which presented outcome data for staff and children together would be considered separately from studies which presented data for staff and students.

### Information sources and search strategy

**Figure 1:**
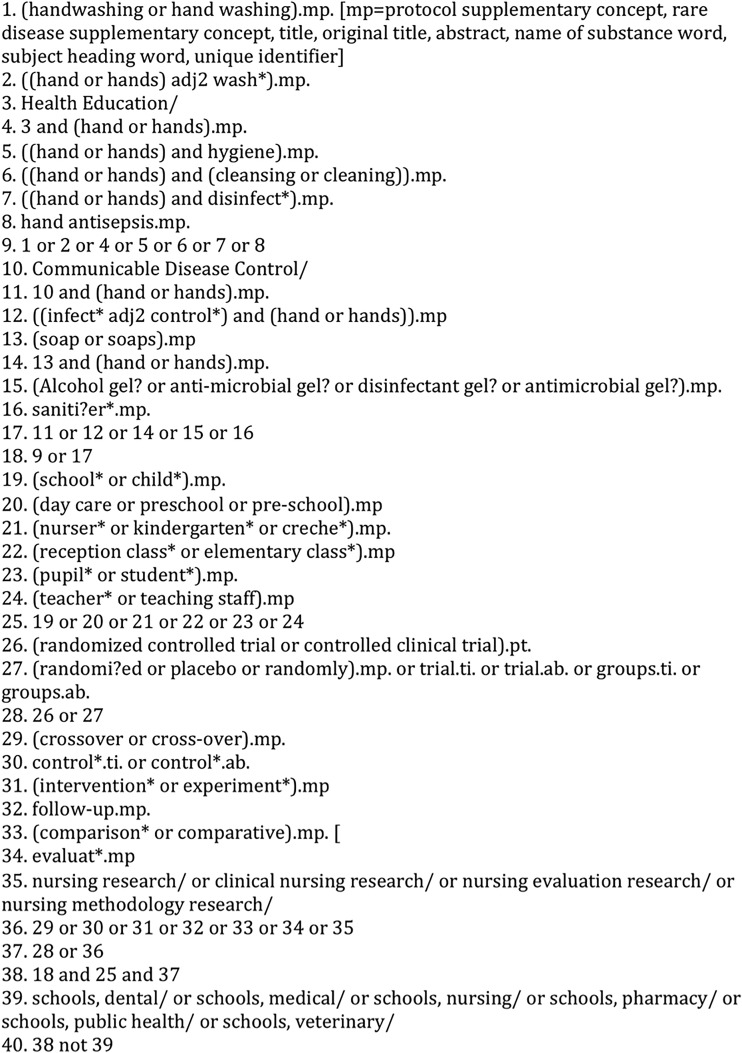
Search strategy used for Medline.

The search strategy had three components: handwashing, population and setting and study type. Handwashing, population and setting terms were extensive; handwashing terms used free-text terms as well as available controlled vocabulary terms. Population and setting terms were not used in education databases (Education Resource Information Center, Australian Education Index, British Education Index). The search focused on sources reporting RCTs and excluded unpublished literature as the coauthors agreed this was unlikely to report RCTs. A broad study type filter was used in databases where RCTs were less well indexed (see figure 1 for MEDLINE search strategy). No date or language restrictions were applied.

Eight electronic databases were initially searched from inception to April 2011: MEDLINE (1950 to date), EMBASE (1980–2011, week 15), Social Science & Science Citation Indexes (ISI Web of Knowledge), CINAHL, Cochrane Library, Education Resource Information Center (1966 to date), Australian Education Index (1979 to date) and British Education Index (1975 to date). The search was updated twice using the same strategy, first to cover up to 26 September 2012, then up to 5 September 2014; dates overlapped with previous searches to ensure items were not missed. Results of each search were uploaded to an EndNote database, combined and deduplicated.

### Study selection and data collection process

All titles were screened for eligibility by one reviewer; 10% were independently screened by a second reviewer (Cohen's κ statistic=≥0.75). Abstracts were independently screened by two reviewers. Where reviewers did not agree, abstracts were included in full paper screening. Full papers were dual reviewed and reasons for exclusion recorded: coauthors moderated where there was disagreement. Additional studies were identified through references in full papers and citation search facilities in ISI Web of Science, journal websites and Google Scholar.

Two potentially eligible abstracts not in English were reviewed by native speakers. A full translation was obtained for the one study that met review criteria.^19^ Protocols for included studies were obtained from trial registers where available.

### Data collection and data items

Two reviewers independently extracted study data using a form developed from a template from another SR^16^ and piloted on a sample of included studies. Data included were: study details; intervention description; study recruitment; random allocation; study baseline data; follow-up; process evaluation; outcomes and analysis. Reviewers discussed differences and recorded moderated results.

### Risk of bias assessment

Study quality was assessed independently by two reviewers using the Cochrane Risk of Bias tool (V.5.1), compliance with reporting guidance^20^ ^21^ and good research practice (research governance, process evaluation, outcome measurement methods) pertinent to interventions with this population in these settings.

### Summary measures

All effect measures pertaining to review outcomes are reported. Where studies included children under 3 years old and stratified the results they presented by age, we only report results for children over 3 years old. Where possible we present unadjusted results, where adjusted results are stated the variables used for adjustment are described. As a large number of studies reported absence by reason, three additional sets of outcome data are presented; absence due to any illness, absence due to RT infection, absence due to GI infection.

### Synthesis of results

We aimed to conduct MAs if studies were sufficiently homogenous and data were adequate. Missing and unclear data were identified in the data extraction form. Studies where additional data could not be accessed were excluded from MA and reasons recorded. Authors were only contacted in exceptional circumstances due to the length of time since completion for many studies. No authors provided additional data. This led to the exclusion of several studies. Six studies were excluded due to design flaws (risk of contamination between study arms); cross-over design,^22^ ^23^ clusters at class level,^24–26^ and clusters at class and school levels.^27^ ^28^ Therefore, MAs were not conducted.

### Additional analyses

Prespecified subgroup analyses (age, gender, location, setting, intervention and duration) and sensitivity analyses were not possible due to poor reporting and data quality.

## Results

### Study selection

**Figure 2:**
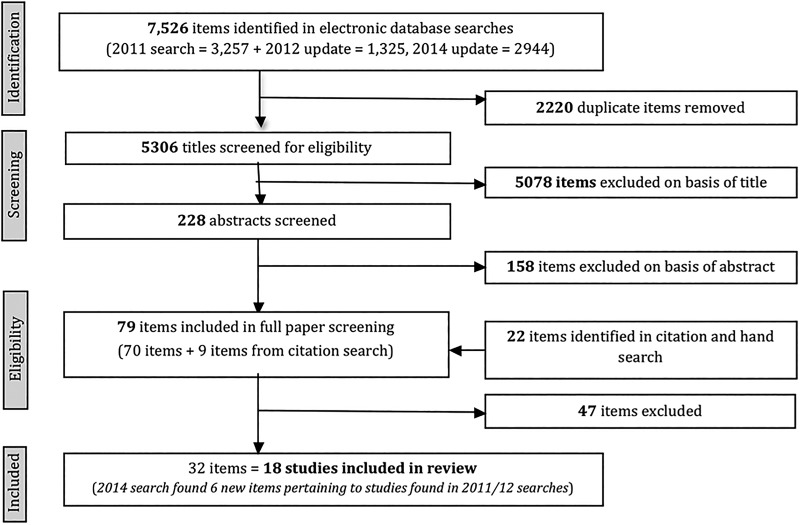
Flow of papers through the review.

Of the 5306 titles assessed for eligibility, 18 studies fitted review criteria (figure 2). Protocols for four RCTs with as yet unpublished results were identified.^29–32^

### Study characteristics

All included studies were cluster RCTs, including two with a cross-over design^22^ ^23^ (table 1).

**Table 1:** Characteristics of included studies

Study author (study name)	Year of study	Population				Intervention (product details provided where reported)	Control (not all authors defined standard practice)	Study design (cluster RCTs)	
		Participants	Age in years (school grade)	Setting	Location			Cluster	Number of clusters
*School-based studies*									
Azor Martínez *et al*^27^ ^28^	2009–2010	School children (n=1640)	4–12 years	Primary school (n=5)	Spain (Almeria)	Handwashing with soap followed by hand sanitiser (ALCO ALOE GEL)	Standard practice	School and classroom	4 schools, 29 classes from another school
Bowen *et al*^33^ (Safeguard Promotion Program)	2003–2004	School children (n=3962)	Median 7.53 years (1st grade)	Primary school (n=90)	China (3 counties in Fujian Province)	(1) Standard programme (teacher training to encourage handwashing with soap, student take home pack) (2) Enhanced programme (standard programme plus supply of safeguard soap, student peer mentors)	Standard practice (Annual statement about Handwashing before eating and after toilet)	School	90 30 intervention (1), 30 intervention (2), 30 controls
Freeman *et al* (WASH programme)^34^ ^35^	2007	School children (n=5989 supplied absence data)	6–16 years; median 13 years (4th–8th grade)	Public primary school (n=135)	Kenya (4 districts in Nyanza Province)	(1) Hygiene promotion (HP) and water treatment (WT) (3 days teacher training, follow-up sessions) (2) HP and WT plus up to 7 new latrines per school	Standard practice	School	135 45 intervention (1) 45 intervention (2) 45 controls
Graves *et al*^36^ (substudy of NICHE: Nyando Integrated Child Health and Education)	2008–2009	School children (precise number not reported)	Age not reported (Students in NICHE study were in 4th–8th grade)	Primary school (n=21)	Kenya (rural western area)	NICHE intervention (multiple components including health promotion by teachers, installation of drinking water, handwashing stations) plus a visual aid poster designed by students in intervention schools	NICHE intervention only	School	21 schools 10 intervention 11 control (14 included in analysis)
Morton and Schultz (Healthy hands)^22^	2000–2001	School children (n=253)	Age not reported (Kindergarten–3rd grade)	Elementary school (n=1)	USA (New England)	Handwashing with soap and AlcoSCRUB alcohol gel use (45 min session for students)	Standard practice (handwashing with soap)	Classroom	17 (*cross-over design*)
Pandejpong *et al*^24^	2009–2010	School children (n=1437)	2–3, 3–4, 4–5, 5–6 years	Private school (n=1)	Thailand (suburban Bangkok)	Application of alcohol hand gel: Two intervention groups (1) every 60 min; (2) every 120 min	Standard practice (alcohol gel application once, before lunch)	Classroom	68 (*not clear how many classes in each arm*)
Pickering *et al*^37^	Unclear	School children (n=1364)	5–10 years (preunit to P5). 1 included a nursery (2–4 years), 4 included 10–13-year-olds (P6-8 grades)	Primary school (n=6)	Kenya (Kibera urban community in Nairobi)	(1) Handwashing with soap. Two soap dispensers installed by toilets, eating area (plus water tank with a spigot). (2) Alcohol-based hand sanitiser use (Purell). Two dispensers installed by toilets, eating area	No intervention (standard practice)	School	6 2 intervention (1) 2 intervention (2) 2 controls
Priest *et al*^38–40^	2009	School children (n=16 245)	5–11 years (school years 1–6)	Primary school (n=68)	New Zealand (Dunedin, Christchurch, Invercargill)	30 min inclass hand hygiene education session, instruction on hand sanitiser use, ‘no touch’ dispensers installed in classrooms	30 min inclass hand hygiene education session only (no instruction on hand sanitiser use)	School	68 schools 34 intervention 34 controls
Sandora *et al*^25^	2006	School children (n=285)	Age not reported (3rd–5th grade)	Elementary school (n=1)	USA (Avon, Ohio)	Handwashing with soap, Aerofirst hand sanitiser use, plus Clorox disinfectant wipes (Student instruction, teachers wiped students’ desks once a day, after lunch)	Standard practice (handwashing with soap)	Team	6 teams in 15 classrooms
Stebbins *et al* (Pittsburgh Influenza Prevention Project)^41–44^	2007–2008	School children (n=3360)	Age not reported (Kindergarten—5th grade)	Elementary school (n=10)	USA (Pittsburgh, Pennsylvania)	Handwashing and Purell hand sanitiser use (45 min presentation for students, educational materials for parents)	Standard practice	School	10 5 intervention 5 controls
Talaat *et al*^45^	2008	School children (n=44 451)	Median 8 years (1st–3rd grade)	Elementary school (n=60)	Egypt (Cairo)	Handwashing with soap (school-specific activities, coordinated by teachers, school nurse; pupils provided soap, drying materials)	Standard practice	School	60 30 intervention 30 controls
Vessey *et al*^23^	Not known	School children (n=383)	Age not reported (2nd and 3rd grades)	Elementary school (n=4)	USA (Butte, Montana)	Hand sanitiser use (one educational session for students)	Handwashing with soap	Classroom	18 (*cross-over design*)
White *et al*^26^	1999	School children (n=769)	5–12 years (Kindergarten—6th grade)	Elementary school (n=3)	USA (California)	Handwashing and alcohol-free hand sanitiser use (all students attended 22-min assembly)	Handwashing and placebo sanitiser use (all students had 22-min assembly)	Classroom	72 32 retained for analysis: 16 intervention, 16 controls
*Non-school based studies*									
Correa *et al*^46^	2008	Children (n=1727)	1–5-years	Child care centre (n=42)	Colombia (6 urban settings)	Purell alcohol-based hand sanitiser use (training workshop for staff and children, monthly refresher workshops)	Standard practice (handwashing with soap)	Child care centre	42 (32 community, 10 preschool)
Ladegaard and Stage^19^	Not known	Children (n=399 aged 3–6 years)	0–2 years and 3–6 years	Nursery (n=8)	Denmark (Borough of Odense)	Handwashing with soap (staff training, take home book, 1 h education session for children)	Standard practice	Nursery	8 4 intervention, 4 controls
Lennell *et al*^47^	2004–2005	Children (n=1477)	0–5 years. Mean: 3.2 years (intervention), 3.1 years (control). Circa 30% <3 years	Day care centre (n=60)	Sweden (10 counties, south and mid-Sweden)	Handwashing with soap and alcohol-based oily disinfectant gel use (instruction, demonstration to staff and children)	Standard practice (handwashing with soap)	Day care centre	60 30 intervention, 30 controls (matched pairs)
Rosen *et al* (Jerusalem handwashing study)^48–52^	2001	Children (n=1029)	3 years and 4 years	Preschool (n=40)	Israel (Jerusalem)	Handwashing with soap (2 3-h staff training sessions, child education programme, take home pack)	Standard practice and alternative take-home pack (about oral hygiene)	Preschool	40 20 intervention 20 controls
Uhari and Möttönen^53^	1991–1992	Children (n=1522)	861 >3 years 661 <3 years Mean: 3.6 years (intervention), 3.5 years (control)	Child day care centre (n=20)	Finland (Oulu city)	Handwashing with soap and alcohol-based oily disinfectant use, plus cleaning environment (staff lecture on infection prevention; cleaning toys; staff encouraged to take sick leave at first sign of symptoms)	Standard practice	Day care centre	20 10 intervention 10 controls (matched pairs)

RCT, randomised controlled trial; WASH, Water, Sanitation and Hygiene.

#### Study participants

Age of participating children was not always reported. Five of the 13 school-based studies included all children in each school;^26^ ^27^ ^37^ ^39^ ^41^ others included one or more age grade. Six studies included children under 3 years.^19^ ^24^ ^37^ ^46^ ^47^ ^53^ These were retained because the interventions included hand hygiene for children as well as staff. Four studies included students over the typical maximum primary school age of 11 years.^27^ ^34^ ^36^ ^37^ These were retained because students’ education level was likely to be equivalent to students in other contexts.

#### Country location and setting

Thirteen studies were school-based; five were in day care facilities or preschools. Institutions were not necessarily representative of settings in that country. For example, one study only included schools with continuous water supply.^45^ Eleven studies were in high-income countries (defined using World Bank categories^54^); only two did not involve hand sanitiser.^19^ ^48^ Four studies were from middle-income countries;^24^ ^33^ ^45^ ^46^ three were from one low-income country (Kenya).^34^ ^36^ ^37^

#### Interventions and comparators

Twelve interventions included hand sanitiser;^22–27^ ^37^ ^39^ ^41^ ^46^ ^47^ ^53^ six focused on handwashing with soap.^19^ ^33^ ^34^ ^36^ ^45^ ^48^ Several interventions included additional infection control measures, such as eliminating shared cups,^48^ water treatment and building new latrines,^34^ ^36^ cleaning toys or equipment.^25^ ^53^ Five included a home component such as parental information.^19^ ^33^ ^41^ ^45^ ^48^

Fourteen studies compared interventions with ‘standard practice’ but this was often unclearly defined. One study was placebo-controlled,^26^ three compared an intervention with an alternative intervention.^23^ ^36^ ^39^ Four studies compared two interventions and a control.^24^ ^33^ ^34^ ^37^ Only two studies adopted a multifactorial design to test the effect of different intervention components.^24^ ^37^

Hand hygiene protocols varied. For example, only 7 of the 12 studies including hand sanitiser described the frequency and/or intensity of use. Nine interventions lasted 10 weeks or less.^19^ ^22^ ^23^ ^25^ ^26^ ^33^ ^34^ ^37^ ^48^

#### Outcomes

The online supplementary table S2 presents study results according to review outcomes. Only three studies^34^ ^36^ ^46^ did not report absence outcomes. Six studies presented results concerning RT infection and/or symptoms;^33^ ^37^ ^41^ ^45^ ^46^ ^53^ four presented results concerning GI infection and/or symptoms.^33^ ^37^ ^46^ ^53^ Two studies reported laboratory results, both pertaining to influenza-like illness (ILI).^41^ ^45^ Six studies presented knowledge, attitude and/or behavioural outcomes.^34^ ^36^ ^37^ ^41^ ^48^ ^53^ No study reported hospital admissions due to infection. Four studies presented staff outcomes.^36^ ^37^ ^48^ ^53^

Outcome definitions and summary measures varied. Three reports did not clearly define illnesses or symptoms.^23^ ^47^ ^48^ Some only reported adjusted outcomes (variables differed between studies).

### Risk of bias within studies

Methodological issues increased risk of bias in most studies (see online supplementary table S1, reviewers’ assessment of the quality and risk of bias of included studies). Some issues highlight difficulties in evaluating behaviour change (eg, lack of participant blinding); others indicate study design weaknesses (eg, random sequence generation) and inadequate reporting (eg, only reported statistically significant results).

Five studies described an adequate method of random sequence generation,^39^ ^41^ ^45^ ^46^ ^53^ only two adequately described allocation concealment.^39^ ^41^ Perhaps unsurprisingly given the nature of the intervention, only the study where a placebo hand sanitiser was the comparator was judged to be at low risk of performance bias.^26^ Only one study^39^ was assessed as having adequately described all measures to blind outcome assessors. The completeness of data reported for each outcome was assessed as adequate in five studies;^23^ ^25^ ^39^ ^46^ ^48^ high risk of selective reporting was identified in four studies.^24^ ^26^ ^37^ ^41^

Four reports did not present baseline data.^19^ ^22^ ^23^ ^26^ Despite being concerned with illness outcomes, only eight reported baseline health data.^24^ ^25^ ^27^ ^39^ ^46–48^ ^53^

Six studies^22–28^ had clusters at class level (two of these applied a cross-over design), therefore increasing risk of contamination between study arms. Not all investigators took clustering into account in sample size calculation or analysis.

Three studies were funded by companies producing hygiene products,^23^ ^25^ ^33^ three used manufacturer-donated products,^22^ ^37^ ^46^ one required parents to provide soap and hand drying materials.^45^ It is unclear whether the way in which these interventions were resourced affected their acceptability, sustainability or study outcomes: only two study reports state the role of these companies in the study, analysis and report. 25 33

Most reports described the intervention protocol and monitoring, three noted intervention costs^24^ ^28^ ^46^ but few presented process evaluation data.

Most outcome measurement methods could have introduced bias due to poor case definition, use of non-validated tools or self-report (including routine school absence reporting data). Some studies which attempted to validate outcomes (eg, illness) experienced attrition due to the complexity of the process (ref. 41, p.3).

### Individual study results

Five of the six studies reporting children's absence and 8 of the 13 studies measuring children's illness absence reported an intervention effect (see online supplementary table S2 for study results according to review outcomes). The one study reporting staff illness absence found it was higher among the intervention group^53^ which may be because the intervention included asking staff not to attend work if they had infection symptoms.

All five studies reporting RT infection incidence showed a reduction, but each applied different outcome definitions. Three reported RT infection symptoms (rhinitis, cough); one^53^ found a reduction in both, one^37^ only identified a reduction in observed rhinorrhoea and another^33^ found no change in cough and a 12% increase in rhinorrhoea episodes (‘standard’ intervention vs control).

Two studies reported GI incidence; one reported a reduction,^46^ the other did not.^53^ Only one of three studies recording diarrhoeal symptoms found any effect.^37^ Two studies reported vomiting outcomes,^37^ ^53^ only one found an effect.^53^

Two studies^41^ ^45^ collecting laboratory results found some evidence of decreased ILI, although in one study this only related to influenza A (ref. 41, Supplemental Digital Content (SDC) 2).

Four of five studies reporting children's behaviour change identified a positive intervention effect.^34^ ^37^ ^41^ ^48^ All five studies reporting changes in children's and/or staff hand hygiene knowledge, attitudes and/or beliefs found an intervention effect.^34^ ^37^ ^41^ ^51^ ^53^

### Synthesis of results

Due to study heterogeneity and the generally low quality of study design and of study reporting, coauthors agreed that it could be misleading to present pooled estimates of the effect of interventions using MAs.

## Discussion

### Main findings

We found 18 cluster RCTs investigating the effect of interventions with a hand hygiene component on absence and infection among 3–11-year-old children in educational settings. Individual study results suggest interventions may reduce children's absence, RT infection incidence and symptoms, and laboratory-confirmed ILI. They may also improve children's and staff hand hygiene attitudes, knowledge and behaviour. Evidence of impact on GI infection or symptoms was equivocal. Despite updating existing SRs and identifying new studies, individual study results appear to show that there remains equipoise about the effectiveness of hand hygiene in preventing RT and GI infection.

### Strengths and limitations of this review

Much has been made of the potential of hand hygiene interventions for reducing infection in this population.^11^ This review provides a more detailed assessment of such interventions and how promising they might be based on studies which apply the most rigorous, RCT evidence. This review updates existing SRs focused on this population, and our comprehensive search strategy resulted in finding more studies than previous SRs. Findings of this review corroborate existing SRs; that studies have significant design limitations and poor quality reporting. The quality of reporting in more recently published studies^27^ ^28^ ^39^ seems to have improved which perhaps indicates the impact of guidance on the reporting of cluster RCTs.^20^ ^21^ This may result in improved evidence, capable of demonstrating the effectiveness of this important public health issue. Despite identifying new studies, it was not possible to produce meaningful MAs (as earlier SRs have found) due to study heterogeneity, study design limitations and poor quality reporting.

Limitations of this SR include that: we assumed that report titles or abstracts would contain ‘handwashing’ or ‘hand/s’ but they did not; unpublished literature was excluded; some included studies had study populations which included children younger and older than the prespecified review age range; RT and GI infection incidence can vary within the age range included in the review, as can the potential effectiveness of interventions (due to children's developmental stage); risk of bias assessment was impeded by inadequate reporting. Furthermore, all interventions with a hand hygiene component were included so the impact of hand hygiene cannot be isolated. This review does not distinguish between handwashing with soap or hand sanitiser use even though these methods may have different resource implications and be differentially effective in eliminating certain pathogens.^55^

### What this study adds

While studies are heterogeneous, there is evidence that hand hygiene interventions among primary school-aged children in educational settings may be beneficial, particularly in reducing RT infection incidence. However, this SR highlights limitations of evidence on this crucial public health issue in a key setting with a vulnerable population and the need for improved studies to enable more definitive assessment (eg, MA) of the effectiveness of simple public health interventions to inform practice. We have four recommendations for future research and which may enable future estimates of the pooled effects of such interventions using MA.

First, better designed and reported cluster RCTs are required. Investigators should apply guidance^20^ ^21^ and learn from robust studies^39^ in order to avoid design flaws (eg, clusters at classroom level) and improve reporting (eg, children's age, control group conditions). Second, studies should incorporate technical advances for outcome measurement, such as the use of environmental swabs to detect the level of viral and/or bacterial contamination in schools^56^ which may enable robust, standardised outcome measures instead of using self-report and observations. Third, research should include process evaluation to refine interventions and establish intervention acceptability and fidelity. Studies which have done process evaluations^40^ ^57^ have identified barriers to hand hygiene including access to adequate sanitary facilities (even in high-income countries), suggesting that provision of hygiene products and education may be insufficient to achieve effective infection prevention and control and more robust studies of complex, multicomponent interventions are required. Fourth, studies should evaluate cost, cost-effectiveness and intervention sustainability in educational settings.

## Conclusion

Interventions to improve hand hygiene in educational settings may reduce RT infection incidence among younger children. More robust, well reported studies are required, especially of multicomponent interventions.

## Supplementary Material

Web supplement

Web supplement
