# Supplementary material for: Effectiveness of hand hygiene interventions in reducing illness absence among children in educational settings: a systematic review and meta-analysis
Source: Arch Dis Child. 2015 Oct 15;101(1):42–50. doi: 10.1136/archdischild-2015-308875 (PMC4717429; doi:10.1136/archdischild-2015-308875)
Supplement: Web supplement [file archdischild-2015-308875-s1.pdf]

Supplementary Data 1: Summary of reviewers' assessment of the quality and risk of bias of included studies

Study	Risk of bias (according to Cochrane Risk of Bias tool)							Additional issues (Cluster RCT method, research governance and ethics, process evaluation, measurement)
	**Random sequence generation**	**Allocation concealment**	**Performance bias**	**Detection bias**	**Attrition bias**	**Reporting bias**	**Justification of bias identified**	
**School-based studies**								
Azor Martinez *et al* 2014(a), 2014(b)	Unclear risk of bias	Unclear risk of bias	High risk of bias	High risk of bias	High risk of bias	Unclear risk of bias	A random number table was used to allocate schools/ classes to each arm but schools were selected because they had previously been involved in a study. Personnel were unlikely to be blind to allocation because they did data collection, visited classrooms and delivered hand hygiene activities. Parents (who reported absence and illness) may not have been blinded. Paediatricians who reviewed medical records of absent pupils and made final diagnosis were not blinded. Diagrams of participant flow in the two study papers show different numbers of participants. A protocol is available; not clear if all outcomes are reported.	Clusters are at two different levels; school and classes. The authors state that they did not adjust for clustering. Observer effect; behaviour might have changed due to presence of researcher/field workers at site. No information on fidelity or adherence to intervention. Authors acknowledge an adverse reaction to the hand sanitizer. There was some baseline information about the use of hand sanitizer at home but only 83% of parents provided this information. Authors state that baseline socio-demographic characteristics of participants were similar.
Bowen *et al.* 2007 (Safeguard Promotion Program)	High risk of bias	Unclear risk of bias	High risk of bias	High risk of bias	Unclear risk of bias	Low risk of bias	Allocation sequence generation was adequately described, but 24 control schools were excluded post randomisation and replaced with non-stratified schools because they distributed the wrong take-home packs. Participants and outcome assessors (teachers) were not blinded but some attempt was made to conceal the aim of the intervention by telling teachers It was a health intervention looking at illness rates among students. It is unclear if outcome data reported (table 4, 5) is complete. A protocol is available; all outcomes are reported.	Adjustment was made for clustering in the sample size calculation and in analysis. Study funders had input into the study protocol but the authors state that they "*were not involved in the study implementation or data analysis*" (p.1168). There are questions about potential contamination and intervention fidelity as the authors reported that some students brought soap from the home pack to use in school. Authors note that there was a lack of sensitivity in the 'health surveillance system' used and there may have been over-reporting of illness (e.g. where the same student was absent twice in one week).
Freeman *et al.* 2012 (WASH programme)	Unclear risk of bias	Unclear risk of bias	High risk of bias	High risk of bias	Unclear risk of bias	Unclear risk of bias	Schools were "*randomly selected and randomly assigned*" (p.382) but it is not clear how. Participants and personnel were not blinded (students reported their own absence) although researchers "*conducted a roll-call assessment of absence for all registered students the day of the field visit to assess the validity of our primary absence measure*" (p.383) a high risk of bias is likely. The flow of participants through the study is unclear. It is unclear whether all outcomes are presented.	Adjustment was made for clustering in the sample size calculation and analysis; ICC is reported. Teachers consented on behalf of students. Absence measure is subject to recall bias (incidence extrapolated from 2-week report given by a sample of students) and "*follow-up data were collected at a time when pupils may have been more likely to attend for test preparation*." (p.389). Also deworming was done in all schools that may have impacted intervention effect. Fewer than 40% of students from intervention arms reported soap was always available for hand washing, suggesting sustainability issues.
Graves *et al* 2011	Unclear risk of bias	Unclear risk of bias	Unclear risk of bias	High risk of bias	High risk of bias	Unclear risk of bias	No description random sequence generation or method of concealment. Not clear whether participants or personnel were blinded. Four trained personnel observed hand	No adjustment for clustering in design or analysis, no ICC reported. Potential for observer effect (behaviour might have changed due to observations). Subjective outcome measures applied (observations

Study	Risk of bias (according to Cochrane Risk of Bias tool)							Additional issues (Cluster RCT method, research governance and ethics, process evaluation, measurement)
	Random sequence generation	Allocation concealment	Performance bias	Detection bias	Attrition bias	Reporting bias	Justification of bias identified	
							hygiene behaviours; they would not have been blind to the presence of the intervention posters. The flow of participants through the study is not clear. This is a sub-study of a larger (NICHE) study; it is not clear whether the outcomes were planned in advance and that all outcomes are reported.	only carried out for two hours in the morning). Possibility of measurement bias - observers estimated some outcomes (e.g. distance between handwashing station and latrine). Authors accept that "*it is not possible to assess the impact of the intervention independent of the physical and educational resources provided by NICHE*" (p.318). Little information on fidelity or adherence but authors report limited access to soap and/or water some sites.
Morton and Schultz 2004 (Healthy hands)	Unclear risk of bias	High risk of bias	High risk of bias	Unclear risk of bias	High risk of bias	Unclear risk of bias	The randomisation method was not clear. This was a crossover study with clusters at the classroom level in the same school, leading to high risk of contamination and performance bias; it would not be possible to conceal the allocation because of the design. The study nurses noted outcome data but were also delivering part of the intervention. There was a higher attrition rate in the 2nd phase; authors suggested this was due to weather changes which may have made children susceptible to dry skin which was exacerbated by the sanitizer. No protocol was identified so it is unclear whether all outcomes are reported.	It is not clear whether adjustment was made for clustering in the sample size calculation and no ICC is reported. McNewar's test for dichotomous variables with paired subjects was used for analysis (p.165). The acceptability of the intervention is questionable during the Winter-time (flu season) as more children experienced dry skin in cold weather. Also, one child felt that the intervention was making her sick.
Pandejpong *et al*. 2012	Unclear risk of bias	Unclear risk of bias	Unclear risk of bias	Unclear risk of bias	Unclear risk of bias	High risk of bias	Authors state they used; "*cluster randomisation to assign the school's classrooms to intervention or control groups*" but do not describe how they did this (p.508); insufficient details about allocation concealment are provided. The study design (clusters at classroom level) introduces potential contamination and performance bias; authors attempted to control for this by having fieldworkers observe compliance with the different time schedules for using the hand gel. It is not clear whether all outcome data are presented; a protocol was not found. It appears that authors only report statistically significant results (p.510).	It is not clear whether adjustment was made for clustering in the sample size calculation, no ICC are reported, but the analysis accounts for clustering. Illness could have been misclassified by parents/guardians. Adherence to the intervention protocol (sanitizer application every 60 or 120 minutes) was monitored and the authors do not explore whether this was sustainable or if the frequency of the application was acceptable to teachers and/or students.
Pickering *et al* 2013	Unclear risk of bias	Unclear risk of bias	High risk of bias	High risk of bias	High risk of bias	High risk of bias	Random sequence generation and allocation concealment are not described. Participants were not blind to allocation as "*the consenting process informed parents of the assignment*" (p.412) and parents could have told children of their allocation. Field researchers were not blinded and it is not clear the outcome assessors were blinded. The flow of	It is not clear whether adjustment was made for clustering in sample size calculation. Analysis methods take clustering into account; ICC are reported. Authors acknowledge that: "*the study was not designed to have sufficient power to detect significant impacts on health*" (p.412). Authors state that "*sanitizer was well-accepted by teachers and students*" but that teachers and students disliked the product odour

Study	Risk of bias (according to Cochrane Risk of Bias tool)							Additional issues (Cluster RCT method, research governance and ethics, process evaluation, measurement)
	**Random sequence generation**	**Allocation concealment**	**Performance bias**	**Detection bias**	**Attrition bias**	**Reporting bias**	**Justification of bias identified**	
							participants through the study is not clear (no diagram presented). Authors clearly state the primary and secondary outcomes and present results for each of these.	before eating (p.416) Authors report there were no adverse events but table 2 presents data suggesting that some participants experienced a skin rash and that "*teachers did report that some students attempted to lick or eat both the sanitizer and liquid soap*" (p.417). Health status and compliance was self-reported.
Priest *et al* (2014)	Low risk of bias	Low risk of bias	High risk of bias	Low risk of bias	Low risk of bias	Low risk of bias	Process of randomisation, allocation concealment and reasons for this are clearly provided by authors. The extent of blinding of participants and researchers is clearly described: participants were not blinded due to the nature of the intervention but investigators not involved in running the trial, outcome assessors and statistician were blind to the group allocation until after the analysis was complete. The flow of schools and individual participants is clearly presented. The trial was registered with a clinical trials registry. Deviations from the planned process and outcomes are set out and explained.	Adjustment was made for clustering in sample size calculation and analysis; ICC are reported. Product formulation is noted. Intervention acceptability, fidelity, adherence and number of skin reactions are reported. Authors report limitations of the study, including that follow up children (for whom reasons for absence were collected) were recruited after clusters were randomised and caregivers knew the allocation. Rate of consent to follow up was low (36.4%) and lower amongst disadvantaged schools. Authors acknowledge potential measurement and recall bias as outcomes were based on caregiver reports. The H1N1 pandemic occurred during the study; some control schools introduced hand sanitizers and all schools may have taken additional preventive steps so there could have been some contamination effect.
Sandora *et al*. 2008	Unclear risk of bias	Unclear risk of bias	High risk of bias	Unclear risk of bias	Low risk of bias	Low risk of bias	The authors describe the randomisation process but it is not clear how teams were assigned and the study was only in one school so participants may have known their allocation although "*the allocation sequence was generated by computer, and teams were assigned to study groups by a study investigator*." (e1556). Due to the nature of the intervention and study design, teachers were likely to know to which study arm they were assigned, although the person receiving parental reports of illness was blind to allocation. A protocol was identified and authors explain missing data and report all pre-specified outcomes.	It is not clear whether adjustment was made for clustering in the sample size calculation, but an ICC is reported. The analysis accounts for clustering but no ICC is reported.<br>The Clorox Company provided the products used in the study.<br>The baseline level of hand sanitizer use in the home was almost 50% (intervention and control groups) suggesting that the intervention was acceptable. Authors note that 63 children refused to participate but it is not clear why. Authors note that they did not observe use of the hand sanitizer so cannot "*address timing of usage in relation to specific exposures*" (e1561), neither can issues of the acceptability of the intervention be ascertained.
Stebbins *et al*. 2011 (Pittsburgh Influenza Prevention Project)	Low risk of bias	Low risk of bias	High risk of bias	High risk of bias	High risk of bias	High risk of bias	Schools were allocated to study arms "*by a constrained randomisation algorithm*" and allocation concealment is described (p.2). Participants were not blinded and not all outcome assessors (teachers) were blinded either. As Stebbins et al note, teachers may have felt pressure to provide "*right*" answers (p.323) in reporting behavioural outcomes. The authors acknowledge high loss to follow up	Adjustment was made for clustering in sample size calculation and analysis, and ICC are reported. The authors indicate that 2 schools used hand sanitizer before which may have affected the outcomes observed. Influenza testing of absent students was only carried out during the flu season that may have distorted results. Authors note adherence to the intervention. However, only results from teachers who responded to all three behavioural outcome surveys were

Study	Risk of bias (according to Cochrane Risk of Bias tool)							Additional issues (Cluster RCT method, research governance and ethics, process evaluation, measurement)
	**Random sequence generation**	**Allocation concealment**	**Performance bias**	**Detection bias**	**Attrition bias**	**Reporting bias**	**Justification of bias identified**	
							and account for this. A protocol is available, but it is not clear if all outcomes are reported.	analysed and the survey may have been subject to reporting and recall bias. The study was underpowered for most outcomes.
Talaat *et al.* 2011	Low risk of bias	Unclear risk of bias	High risk of bias	High risk of bias	Unclear risk of bias	Unclear risk of bias	Random sequence generation described but it is not clear whether allocation was concealed. Participants and outcome assessors (included teachers) were not blinded; authors note underreporting of illness as a cause for absenteeism in intervention schools. Lack of precise description of outcomes means it is difficult to assess level of reporting bias. Authors do not reflect on the loss of data caused by parents declining consent for their children's swab specimens to be taken. No protocol identified.	Adjustment was made for clustering in the sample size calculation and analysis; no ICC were reported. Absence incidence may have been overestimated if a child were ill at the end of 1 week and at the beginning of the next, although this is could have been the same in intervention and control schools. Also, the rapid test used for influenza diagnosis had low sensitivity and there was a low rate of testing in students absent due to ILI in control schools compared to intervention schools. Monitoring teams found that approximately 93% of students were observed to have soap and drying material available.
Vessey *et al.* 2007	Unclear risk of bias	High risk of bias	High risk of bias	High risk of bias	Low risk of bias	Unclear risk of bias	Insufficient information on randomisation was provided. This was a crossover study with clusters at the classroom level in the same school so there is high risk of performance bias and it would difficult to conceal allocation. Authors note teachers were more critical about reporting children to the school nurse during the study because they were not blinded. School secretaries collected absence information but are likely to have known the classes receiving the intervention. Authors report loss to follow-up. No protocol was identified so it is unclear whether all pre-specified outcomes are reported.	It is not clear whether adjustment was made for clustering in the sample size calculation or analysis. No ICC are reported. A hand sanitizer manufacturer funded the study and whilst it was not found to be more effective than normal practice in preventing illness absence, the authors present data showing teachers preferred the sanitizer and perceived "*improved adherence*" to hand sanitizer than hand washing, although teachers also noted when the sanitizer dripped it "*removed the wax from the tile*" (p.371). Authors noted it might be difficult to maintain supplies of soap, towels and hand sanitizer, and limitations of absenteeism as a proxy measure and parent reports (p.371).
White *et al*. 2001	Unclear risk of bias	Unclear risk of bias	Low risk of bias	High risk of bias	High risk of bias	High risk of bias	Randomisation and allocation concealment processes are unclear. There is low risk of performance bias as this was a placebo-controlled trial. Teachers assessed outcomes and were blind to allocation but the measure used was subjective. Authors report a large loss to follow up due to lack of compliance with the intervention (classes which did not comply with minimum product use of ≥3 times per day were excluded from analysis). No protocol was identified; pre-specified outcomes are not clearly presented.	It is unclear whether adjustment was made for clustering in the sample size calculation, no adjustment was made in analysis; no ICC are reported. Intervention acceptability is questionable because authors admit that teachers were "*tired of the study*" and not all complied with the intervention – 40 classes did not meet the 'minimum' required product use of ≥3 times per day (p.262-3).
**Non-school based studies**								
Correa *et al*. 2012	Low risk of bias	Unclear risk of bias	High risk of bias	High risk of bias	Low risk of bias	Low risk of bias	Random sequence generation was thoroughly described (p.478); allocation concealment was not. Participants, study personnel (teachers) and outcome assessors were not blinded (p.478). Authors account for attrition and state how	Adjustment was made for clustering in the sample size calculation and analysis; ICC are reported. Authors attempted to reduce ascertainment bias by not providing teachers with case definitions and case registry were reviewed by project coordinator who was blinded

Study	Risk of bias (according to Cochrane Risk of Bias tool)							Additional issues (Cluster RCT method, research governance and ethics, process evaluation, measurement)
	**Random sequence generation**	**Allocation concealment**	**Performance bias**	**Detection bias**	**Attrition bias**	**Reporting bias**	**Justification of bias identified**	
							many children and centres were lost to follow up. However "*after trial onset, 372 new children entered trial centers*" (p.478-9). A protocol is published and stated outcomes were reported.	to study arms. Intervention adherence was not reported, but authors suggest it was acceptable as in 7 centres, hand sanitizer use amongst teachers almost replaced hand washing when hands were not soiled.
Ladegaard and Stage 2009	Unclear risk of bias	Unclear risk of bias	High risk of bias	Unclear risk of bias	Unclear risk of bias	Unclear risk of bias	Authors describe a random component to the sequence generation (drawing lots) but it is not clear who did this and whether allocation was concealed. There is little discussion of participant blinding or outcome assessment, but it is likely that participants were not blinded due to the nature of the intervention. Insufficient information was provided to assess attrition or reporting bias.	It is unclear whether adjustment was made for clustering in the sample size calculation and analysis; no ICC are reported.<br>Authors note that staff found it difficult to refuse entry to children who were unwell at arrival and during observation, it was noted that hygiene guidelines and hand washing facilities were not always maintained, suggesting issues of intervention acceptability.
Lennell *et al*. 2008	Unclear risk of bias	Unclear risk of bias	High risk of bias	High risk of bias	High risk of bias	Unclear risk of bias	Insufficient information to judge randomisation or allocation concealment. Participants and study nurses were not blind to allocation: "*because it was not possible to produce a control gel with the same characteristic smell of the disinfectant gel*" (p.1674). Outcome data were sent away for processing but nurses collected sickness absence data and sought missing data. Centres that did not provide adequate attendance information were excluded from analysis (31/60 centres); children in excluded centres differed from those that were retained (p.1678). Authors state that they will measure the outcome using parental data on attendance but results presented use staff data.	Adjustment was made for clustering in the sample size calculation and may have been carried out for analysis; no ICC are reported. There were issues concerning intervention adherence as some children followed the hand washing protocol but did not apply the alcohol gel. It is likely that there was reporting bias as "*parents alone made the decision whether their child was absent from DCC due to* illness" (p.1673). There is also the possibility of recall bias as reason(s) for absence were collected monthly. The method for outcome measurement changed from parent report to use of routine data.
Rosen *et al*. 2006 (Jerusalem hand washing study)	Low risk of bias	Unclear risk of bias	Unclear risk of bias	Unclear risk of bias	Low risk of bias	Low risk of bias	Random sequence generation is described but allocation concealment is not adequately described. "*educators, parents and field research staff were… not told that the study included 'intervention' and 'control' groups and that they were being compared with respect to hand washing behaviour and absenteeism*" but risk of bias is unclear because field staff who assessed outcomes may have broken this blinding, as they: "*sometimes became aware that the program was being run in a certain preschool*" (p.28). Explanations for missing data are provided. There is a published protocol; authors report on all outcomes stated in the protocol.	Adjustment was made for clustering in the sample size calculation and analysis; an ICC is reported. Authors indicate that participants were not told that they were being assigned to an intervention and control group which raises ethical issues about informed consent. Authors note that there may have been contamination due to proximity of preschools. Educators were accepted on a *'first to agree, first to be accepted'* basis (p.379) which may have introduced selection bias. There is likely to have been contamination as only 82% of participants received the correct take-home pack and the authors state that they "*received reports of some children exchanging videos, and of other inviting friends and relatives to view the video in their homes*" (p.383).

**Study**	**Risk of bias (according to Cochrane Risk of Bias tool)**							**Additional issues (Cluster RCT method, research governance and ethics, process evaluation, measurement)**
	**Random sequence generation**	**Allocation concealment**	**Performance bias**	**Detection bias**	**Attrition bias**	**Reporting bias**	**Justification of bias identified**	
Uhari and Möttönen 1999	Low risk of bias	Unclear risk of bias	High risk of bias	High risk of bias	Unclear risk of bias	Unclear risk of bias	Authors report random sequence generation but not how allocation concealment was achieved. Participants, study personnel and parents were not blinded. No protocol was identified; there was insufficient description of pre-specified outcomes or participant flow to assess risk of bias.	It is not clear whether adjustment was made for clustering in sample size calculation or analysis (no ICC are reported). Authors note potential for contamination as; "*some families* [had] *one child at an intervention CDCC and another at a control CDCCs, and some of the personnel changed their working place between intervention and control CDCCs during the trial*". Study nurses estimated intervention compliance which may have introduced bias.
