# Supplementary material for: Effectiveness of hand hygiene interventions in reducing illness absence among children in educational settings: a systematic review and meta-analysis
Source: Arch Dis Child. 2015 Oct 15;101(1):42–50. doi: 10.1136/archdischild-2015-308875 (PMC4717429; doi:10.1136/archdischild-2015-308875)
Supplement: Web supplement [file archdischild-2015-308875-s2.pdf]

Supplementary Data 2: Summary of study outcomes corresponding to review outcomes

- Outcomes presented are selected from study reports to best fit the review outcomes; study authors may present other results, too.
- Results relate to children, not staff in educational settings or family members/caregivers unless otherwise stated.
- Where authors present them separately, only results pertaining to children > 3 years old are presented here.
- * denotes a school-based study
- ILI - Influenza-like Illness

Study	Study outcome(s) presented	Results (effect of the intervention) and author conclusions
**Review outcome (a) reduction in rate or change in respiratory infection**		
Bowen *et al* 2007*	In-class illness incidence due to upper respiratory tract infection (URTI)	Standard program: Median average of 0.38 episodes per 100 student weeks in the intervention group, a 21% decline compared to the control group (0.48 episodes per 100 student weeks), p>0.4 (table 4, p.1169)<br>Enhanced program: Median average of 0 episodes per 100 student weeks in the intervention group, a 100% decline compared to the control group (0.48 episodes per 100 student weeks), p=0.21 (table 4, p.1169)
Correa *et al* 2012[1]	New cases of acute respiratory infection	Unadjusted incidence density: 2.18 per child-year; 2.06 per child-year (intervention) vs 2.28 per child-year (control) p = 0.0163. (ICC 0.01). (p.480)
Stebbins *et al* 2011*	Total ILI during intervention	Unadjusted incidence rate ratio 0.86 (95% CI 0.57, 1.28) p = 0.45 (ICC 0.01). Adjusted IRR 0.86 (95% CI 0.60,1.22), p = 0.41 (Table SDC 2 – adjusted for percent students receiving subsidized lunch, student race, grade, class size)
Talaat *et al* 2011*	Laboratory-confirmed in-class influenza episodes	The rate of lab-confirmed influenza was higher among students who reported their illness in control schools (35%) than the rate in intervention schools (18%) (p<0.01).
Uhari and Möttönen 1999[2]	Episodes of infection due to rhinitis (children >3 years)<br>Episodes of infection due to cough (children >3 years)	2.7 episodes per person year at risk (intervention), vs 3.1 per person year at risk (control); a 13% (95% CI 3, 23) difference (p = 0.003).<br>2.5 episodes per person year at risk (intervention), vs 2.6 per person year at risk (control); a 4% (95% CI -8, 15) difference (p = 0.49). (Table 3)[*translated*]
**Review outcome (b) reduction in rate or change in signs and symptoms of respiratory infection**		
Bowen *et al* 2007*	Rates of in-class illness (rhinorrhoea)<br><br><br><br><br><br>Rates of in-class illness (cough)	Standard program: Median average 0.19 episodes per 100 student weeks in intervention group, a 12% increase compared to the control group (median 0.17 episodes per 100 student weeks), p>0.4 (table 4, p.1169).<br>Enhanced program: Median average 0 episodes per 100 student weeks in intervention group, a 100% decrease compared to the control group (median 0.17 episodes per 100 student weeks), p=0.30 (table 4, p.1169).<br>Standard program: Median average of 0.08 episodes per 100 student weeks in intervention group, a 0% difference compared to the control group (0.08 episodes per 100 student weeks), p>0.4 (table 4, p.1169).<br>Enhanced program: Median average of 0 episodes per 100 student weeks in intervention group, a 100% decline compared to the control group (0.08 episodes per 100 student weeks), p=0.25 (table 4, p.1169)
Pickering *et al* 2013*	Self-reported cough<br><br><br>Observed rhinorrhoea	Sanitizer vs. control, Risk ratio (RR) =0.89 (95% CI 0.775-1.05, p=0.16)<br>Soap vs. control, RR=1.03 (95% CI 0.88-1.21, p=0.73)<br>Sanitizer vs. soap, RR=0.86 (95% CI 0.73-1.01, p=0.07)<br>Sanitizer vs. control, RR=0.77 (95% CI 0.62-0.95, p=0.02)<br>Soap vs. control, RR=0.77 (95% CI 0.62-0.95, p=0.01)<br>Sanitizer vs. soap, RR=1.00 (95% CI 0.84-1.18, p=0.99) (table 3, p.415)
Uhari and Möttönen 1999	Rhinitis (children >3 years)<br><br><br>Cough (children >3 years)	28.1 events per person year at risk (intervention), compared to 35.3 per person year at risk (control); a 20% (95% CI 18, 23) difference between the two groups (p = 0.001).

[1] The study authors state that: "Incidence densities… were calculated as number of new cases divided by number of susceptible child-days at risk" (Correa et al 2012, p.479). Incidence density can be defined as: "the ratio of incident cases to the population at risk in the course of a time period" (Philippe 2000) and differs from cumulative incidence in that it measures the intensity of a behaviour in a setting whereas cumulative incidence measures the frequency of people doing that behaviour in a setting. Reference: Philippe, P (2000) *Density Incidence And Cumulative Incidence: A Fundamental Difference*. The Internet Journal of Internal Medicine 2(2).

[2] Uhari and Möttönen also report episodes of infection amongst personnel by infection type.

Study	Study outcome(s) presented	Results (effect of the intervention) and author conclusions
		25.0 events per person year at risk (intervention), compared to 26.9 per person year at risk (control); a 7% (95% CI 4, 10) difference between the two groups (p = 0.001). (table 2) [*translated*]
**Review outcome (c) reduction in rate or change in GI infection**		
Correa *et al* 2012[1]	New cases of acute diarrheal diseases	Unadjusted incidence density: 0.75 per child-year; 0.61 per child-year (intervention) vs 0.88 per child-year (control) p < 0.0001 (ICC 0.004) (p.480)
Uhari and Möttönen 1999	Episodes of infection due to diarrhoea (children >3 years)	0.4 episodes per person years at risk (intervention) vs. 0.4 per person year at risk (control); 0% difference (95% CI 18, 25) p = 0.59.
	Episodes of infection due to vomiting (children >3 years)	0.7 episodes per person years at risk (intervention) vs. 0.9 per person year at risk (control); 22% difference (95% CI 6, 33) p = 0.008.(table 3)[*translated*]
**Review outcome (d) reduction in rate or change in signs and symptoms of GI infection**		
Bowen *et al* 2007*	In-class illness incidence due to diarrhoea	Standard program: Median average 0 episodes per 100 student weeks (intervention) vs. 0 episodes per 100 student weeks (control), p>0.4 (table 4, p.1169). Enhanced program: Median average 0 episodes per 100 student weeks (intervention) vs. 0 episodes per 100 student weeks (control), p>0.4 (table 4, p.1169).
Pickering *et al* 2013*	Diarrhoea symptoms (3+ loose/watery stools in 24 hours)	Sanitizer vs. control, Risk Ratio (RR)=0.75 (95% CI 0.52-1.10, p=0.14). Soap vs. control, RR=0.84 (95% CI 0.58-1.22, p=0.36). Sanitizer vs. soap, RR=0.89 (95% CI 0.61-1.30, p=0.56)
	Diarrhoea (any loose/ watery stool in 24 hours)	Sanitizer vs. control, RR=0.87 (95% CI 0.72-1.04, p=0.12). Soap vs. control, RR=1.09 (95% CI 0.92-1.30, p=0.33). Sanitizer vs. soap, RR=0.80 (95% CI 0.67-0.95, p=0.01)
	Diarrhoea (loose/ watery stool identified on stool chart)	Sanitizer vs. control, RR=0.87 (95% CI 0.70-1.08, p=0.19). Soap vs. control, RR=1.04 (95% CI 0.85-1.29, p=0.69). Sanitizer vs. soap, RR=0.83 (95% CI 0.67-1.03, p=0.09)
	Vomiting	Sanitizer vs. control, RR=0.69 (95% CI 0.44-1.09, p=0.11). Soap vs. control, RR=0.95 (95% CI 0.62-1.46, p=0.81). Sanitizer vs. soap, RR=0.93 (95% CI 0.53-1.63, p=0.80)
Uhari and Möttönen 1999	Diarrhoea (children >3 years)	1.1 events per person year at risk (intervention) compared to 1.1 per person year at risk (control); 0% difference (95% CI -17, 18) between intervention and control group (p = 0.86).
	Vomiting (children >3 years)	1.1 events per person year at risk (intervention), compared to 1.5 per person year at risk (control); 27% difference (95% CI 20, 40) between the two groups (p = 0.001). (table 2) [translated]
**Review outcome (e) reduction in rate or change in absence**		
***Absence only*[3]**		
Azor Martinez *et al* 2014*	Absence (any reason)	Academic year 2009-10: Incidence of episodes/100 children/day Relative Risk (RR) = 1.115 (95% CI 1.105-1.2, p<0.001). Percent total absent days RR = 1.06 (95% CI 1.03-1.10, p<0.001).<br>During influenza season: Incidence of episodes/100 children/day RR = 1.22 (95% CI 1.13-1.32, p<0.001). Percent total absent days RR = 1.08 (95% CI 1.01-1.14, p<0.015) (table 2, p.635)
Freeman *et al* 2012*	Pupil-reported school absence	Adjusted odds ratio (standard intervention vs. control): 0.81 (95% CI 0.50,1.35), p = 0.43 (standard intervention + sanitation vs. control: OR 0.97 95% CI 0.55,1.69, p = 0.90) (2012, p.386, table 4, p.387) (adjusted to account for clustering of students within schools and stratification of geographical districts, p.383).
Priest *et al* 2014*	Number of absence episodes for any reason - all children	Incidence Rate Ratio (hand sanitizer vs. control) = 0.94 (95% CI 0.84,1.05; p=0.283) (table 4, p.11)
Rosen *et al* 2006	Overall absenteeism for any reason	Adjusted relative risk 1.00 (CI 0.90, 1.14), p = 0.97 (2006, table 3, p.30) (adjusted for baseline value, educational sector; Rosen et al. 2006, p.381)
Stebbins *et al.* 2011*	Total absences during intervention	Unadjusted Incidence Rate Ratio 0.81 (95% CI 0.60, 1.10), p = 0.18. ICC 0.02 (Adjusted IRR 0.74 [95% CI 0.56, 0.97], p= 0.03) (table SDC2 – adjusted for percent students receiving subsidized lunch, student race, grade, class size)
White *et al*. 2001*	Absence incidence	"*Absence incidence in the study group was approximately 33.8% (p <.01) lower than the control group*" (p.262)

[3] Uhari and Möttönen also report parental absence from work due to child's illness.

Study	Study outcome(s) presented	Results (effect of the intervention) and author conclusions
Absence due to any illness		
Azor Martinez *et al* 2014*	Absence due to respiratory illness, GI or ILI	Academic year 2009-10: Episodes/100 children/day Relative Risk (RR)= 1.59 (95% CI 1.46-1.74, p<0.001). Percent total absent days RR = 1.46 (95% CI 1.37-1.55, p<0.001). During influenza season: Episodes/100 children/day RR = 1.49 (95% CI 1.29-1.71, p<0.001). Percent total absent days RR = 1.35 (95% CI 1.23-1.48, p<0.001)
Bowen *et al* 2007*	Absence incidence	Standard program: Median average 1.15 episodes per 100 student weeks (p=0.08, 44% decline) in intervention vs. 2.04 episodes per 100 student weeks in control (table 5, p.1170). Enhanced program: Median average 1.19 episodes per 100 student weeks (p=0.03, 42% decline) in intervention vs. 2.04 episodes per 100 student weeks in control (table 5, p.1170).
Ladegaard and Stage 1999	Average number of days absent due to illness (3-6 year olds)	Intervention group: average number days absent due to illness fell from 3.06 days (observation period) to 2.53 (intervention period) and 1.90 days (outcome period). Control group: average number days absent fell from 2.94 days (observation period) to 2.20 days (intervention period) then rose to 2.71 (outcome period). (table 2).
Lennell *et al* 2008	Rate of absenteeism due to infections	Unadjusted Incidence Rate Ratio: 0.86 (95% CI 0.78,0.94) (p.1678)<br>Adjusted IRR 0.88 (95% CI 0.80, 0.96) (table 2, p.1678 – adjusted for age, number of hours/week at day care centres, asthma or allergies)
Morton and Schultz 2004*	Number of absences due to infectious illness	"*Using McNewar's test for dichotomous variables with paired subjects, significantly fewer children became ill while using alcohol gel as an adjunct to regular hand washing than when using regular hand washing only (chi square = 7.787; p = .0053). The odds of being absent due to infectious illness were reduced by 43% with adjunct use of alcohol gel*." (p.165)
Pickering *et al* 2013*	School absence due to illness	Fewer students (11%) in sanitizer intervention schools reported missing at least 1 day of school because of illness in the prior week compared with students at control schools (OR = 0.51, SE = 0.1, P < 0.01). Students in hand washing intervention schools also reported lower rates (14%) of illness-related absenteeism at follow-up than students at control schools, but the difference was not significant (OR = 0.66, SE = 0.3, P = 0.37). (p.416)
Priest *et al* 2014*	Number of absence episodes due to any illness	Incidence rate ratio (hand sanitizer vs. control) = 1.06 (95% CI 0.94,1.18; p=0.346) ICC 0.018 (95% CI 0.012,0.043) (Table 4, p.11)
Rosen *et al* 2006	Illness absenteeism	Adjusted Relative Risk 1.00 (CI 0.81,1.32), p = 0.97 (2006, p.30 and table 3). (Adjusted for baseline value, educational sector; Rosen et al. 2006, p.381)
Stebbins *et al* 2011*	Absence due to any illness during intervention	Unadjusted Incidence Rate Ratio 0.77 (95% CI 0.41, 1.45), p=0.42<br>Adjusted IRR 0.75 (95% CI 0.49, 1.16), p=0.20 (adjusted for percent students receiving subsidized lunch, student race, grade, class size)
Talaat *et al* 2011*	Absence caused by overall illness	Number of episodes: 13,247 (intervention), 19,094 (control); a 21% reduction in illness absence (p<0.0001) (table 2)
Uhari and Möttönen 1999	Child absence due to illness<br><br><br>Personnel absence due to illness	"*In 8 of the 10 pairs of Child Day Care Centres, the proportion of days that children were absent because of illness was less in intervention centres, this difference being statistically significant [p< 0.03(fig 1)]*."<br>"Despite the reduced number of infections, *the personnel of the intervention day care centres had more days of absence due to infections than personnel in the control centres, 5.3 vs. 4.6 per PYR, a 15% increase (95% CI 7%,26%, p < 0.001)*." [translated]
Vessey *et al* 2007*	Illness-related absenteeism	Two-tailed t-test of mean differences of number of days absent between intervention (mean average number days absent: 26.77 days, SD 7) and control (mean average number days absent: 25.44 days, SD 10.27) = 0.664 (df 34), showing no significant difference between groups (table 1, p.371).
White *et al*. 2001*	Illness absence incidence	Relative risk 0.67 (CI not reported). (p.263, table 4). "*Absence incidence in the study group was approximately 33.8% (p< .001) lower than in the control group*" (p.262)
Absence due to respiratory infection		
Azor Martinez *et al* 2014*	Absence due to ILI	During influenza season: Incidence of episodes/100 children/day Relative Risk (RR): 2.50 (95% CI 1.73-3.62, p<0.001). Percent total absent days RR: 2.64 (95% CI 2.16-3.21, p<0.001) (table 3, p.635)

Study	Study outcome(s) presented	Results (effect of the intervention) and author conclusions
Bowen *et al* 2007*	Absence due to URTI (upper respiratory tract infection)	Standard program: Median average of 0.43 episodes per 100 student weeks (intervention); a 39% decline compared to control (0.70 episodes per 100 student weeks), p = 0.34 (table 5, p.1170).<br>Enhanced program: Median average of 0.48 episodes per 100 student weeks (intervention); a 31% decline compared to control (0.70 episodes per 100 student weeks), p = 0.33 (table 5, p.1170).
Ladegaard and Stage 1999	Number of days absent due to bronchitis/pneumonia (3-6 year olds)	Intervention: number of days absent fell from 7 days (observation period) to 2 days in the intervention and outcome periods. In the control group, number of days absent declined from 9 days (observation period) to 5 days (intervention period) to 2 days in the outcome period. (table 3).
Morton and Schultz 2004*	Number of absences due to respiratory or GI infection	"*Significantly fewer children in the alcohol gel group (n=39) contracted a respiratory or GI illness than in the control group (n=69)*." (p.166) [*Note: results not separately presented for RT and GI illness*]
Pandejpong *et al* 2012*	Change in the rate of absence caused by physician-confirmed ILI	"*absenteeism rate due to confirmed ILI was significantly higher in the control group (0.026) compared with intervention (1) (0.017) (rate difference 0.0096; 95% CI, 0.004-0.016; P= .002) and also in the intervention (2) (0.026) compared with intervention (1) (rate difference 0.009; 95% CI, 0.002-0.015; P= .008). No significant difference was found between intervention (2) group and the control group (rate difference, 0.001; 95% CI, 0.005-0.007; P=0.743).*"(p.509).
	Change in the rate of absence caused by total reported ILI (with and without physician confirmation)	"*rates of absenteeism from ILI both with and without a doctor's confirmation were 0.069 in the intervention (1) group, 0.065 in the intervention (2) group and 0.070 in the control groups. No significant effect was found across rates*." (p.509)
Priest *et al* 2014*	Number of absence episodes due to respiratory illness - follow up children only	Incidence Rate Ratio (hand sanitizer vs. control) = 1.05 (95% CI 0.92,1.20; p=0.439) ICC 0.015 (95% CI 0.011,0.037) (Table 4, p.11)
Sandora *et al.* 2008	Rate of absence caused by respiratory infection	Unadjusted rate ratio was 1.07 (95% CI: 0.92, 1.24, p=0.39).<br>Adjusted rate ratio was 1.10 (95% CI: 0.97,1.24, p=0.12) (p.e1559 – adjusted for race, health status, family size, current hand sanitiser use in the home)
Stebbins *et al* 2011*	Cumulative incidence of absence episodes associated with influenza B	Adjusted Incidence Rate Ratio: 0.81 (95% CI: 0.54, 1.23), P = 0.33
	Cumulative incidence of absence episodes associated with influenza A	Adjusted Incidence Rate Ratio: 0.48 (95% CI: 0.26, 0.87), P < 0.02
	Cumulative incidence of absence episodes associated with influenza B.	Adjusted Incidence Rate Ratio: 1.45 (95% CI: 0.79, 2.67), P = 0.23 (p.4) (Adjusted for percent students receiving subsidized lunch, student race, grade, class size)
Talaat *et al* 2011*	Incidence of absence due to ILI	"*In control schools, 65.5% (n=1,671) of students were absent caused by ILI… In the intervention schools, ILI was responsible for 53.7% (n=917) of absenteeism*" A reduction of 40%, p=<0.0001 (table 2, table 2).
White *et al*. 2001*	Total respiratory-related absence<br>Respiratory illness absence incidence	"*Total respiratory-related absences decreased by 30.3% (p<.001) in the study group, compared with control* [placebo] *group. Similar decreases in respiratory-related absence-incidences were observed in the study group by 31.7% (p<.01) as compared with the placebo group*." (p.262)
Absence due to GI		
Azor Martinez *et al* 2014*	Absence due to Acute Gastroenteritis	Bivariate analysis: Incidence Rate Ratio (IRR): 0.65 (95% CI 0.54-0.79, p< 0.001).<br>Multiple regression analysis: Adjusted IRR: 0.64 (95% CI: 0.52-0.78, p<0.001) (e36) (Adjusted by sex, immigrant, age, father's/mother's profession, family size, dwelling type, previous hand sanitiser use in the home, correct handwashing, acute-gastroenteritis preventive behaviours, table 2, e38)
Bowen *et al* 2007*	Absence due to diarrhoea	Median 0 episodes per 100 student weeks in standard intervention group, expanded intervention group and control group (table 5, p.1170)
Ladegaard and Stage 1999	Number of days absent due to diarrhoea	Among 3-6 year olds in intervention group, the number of days absent increased from 15 days (observation period) to 23 (intervention period) then fell to 7 days (outcome period). The number of days absent in the control group increased from 21 days (observation period) to 23 days (intervention period) to 16 days in the outcome period. (table 3).

Study	Study outcome(s) presented	Results (effect of the intervention) and author conclusions
Morton and Schultz 2004*	Number of absences due to GI infection	"*Significantly fewer children in the alcohol gel group (n=39) contracted a respiratory or GI illness than in the control group (n=69)*." (p.166) [*Note: results not separately presented for RT and GI illness*]
Priest *et al* 2014*	Number of absence episodes due to GI - follow up children only	Incidence Rate Ratio (hand sanitizer vs. control) = 1.11 (95% CI 0.82,1.52; p=0.490) ICC 0.027 (95% CI 0.023,0.066) (Table 4, p.11)
Sandora *et al.* 2009	Rate of absence caused by GI illness	Unadjusted rate ratio: 0.86 (95% CI: 0.79, 0.94, p<.01).<br>Adjusted rate ratio: 0.91 (95% CI 0.87,0.94, p < .01) (p.e1559 – adjusted for race, health status, family size, current hand sanitiser use in the home)
Talaat *et al*. 2011*	Incidence of absences due to diarrhoea	639 episodes in intervention, compared to 1,316 in control; a 33% reduction in absences due to diarrhoea, p=< 0.0001 (table 2)
White *et al*. 2001*	Total GI-related absence<br>GI illness absence incidence	"T*otal GI-related absences were decreased by 32.8% (p<.01) in the study group, compared with the control* [placebo] *group. Similar decreases in gastrointestinal absence-incidences were observed in the study group by 38.6% (p<.01) as compared with the placebo group*." (p.262)
**Review outcome (f) Laboratory results of respiratory and/or GI infection**		
Stebbins *et al* 2011*	Absence due to episodes of laboratory confirmed influenza (A and/or B)<br>Absence due to episodes of laboratory confirmed influenza A<br>Absence due to episodes of laboratory confirmed influenza B	Unadjusted Incidence Rate Ratio: 0.94 (95% CI 0.59, 1.52), p = 0.81 (ICC 0.001). Adjusted Incidence Rate Ratio: 0.81 (95% CI 0.51, 1.23), p = 0.33.<br>Unadjusted Incidence Rate Ratio 0.58 (95% CI 0.31, 1.10), p = 0.10 (ICC 0.002). Adjusted Incidence Rate Ratio 0.48 (95% CI 0.26, 0.87), p = 0.02<br>Unadjusted Incidence Rate Ratio 1.60 (95% CI 0.91, 2.84), p = 0.11 (ICC <0.001). Adjusted Incidence Rate Ratio 1.45 (95% CI 0.79, 2.67), p = 0.23<br>(All adjusted for percent students receiving subsidized lunch, student race, grade, class size, SDC 2)
Talaat *et al**	Incidence of laboratory-confirmed influenza (in-class and absence).	Intervention group: 125/808 cases tested (in-class and absent) were positive for influenza; compared to 795/1075 cases tested (in-class and absent) from control. "*laboratory confirmed influenza reduced 50% (p<0.0001)*" (p.1)
**Review outcome (g) Behaviour change related to hand hygiene**		
Graves *et al* 2011*	Proportion of students washing hands after latrine use	Difference in proportion of students washing hands was not significant; 0.06 (95% CI -0.27, 0.38). Comparing baseline to follow-up the proportion of students washing hands increased by 2.7% in control schools and decreased by 2.7% in intervention schools (p.314)<br>Hand washing behaviour was not significantly associated with distance of the hand washing station from the latrine, visibility from the classroom or visibility from the latrine (p.314).
Freeman *et al* 2012*	Student WASH practices	Percent of students who reported washing hands after using a latrine: Intervention (1) 78% (SE=5) at baseline, 87% (SE=2) at follow up (p=0.11); Intervention (2) 83% (SE=5) at baseline, 89% (SE=5) at follow up (p=0.18); Control 82% (SE=3) at baseline, 81% (SE=3) at follow up.<br>Percent of students who used soap in the hand washing demonstration: Intervention (1) 71% (SE=5) at baseline, 78% (SE=7) at follow up (p=0.75); Intervention (2) 85% (SE=3) at baseline, 81% (SE=8) (p=0.62) at follow up; Control 82% (SE=5) at baseline and 84% (SE=3) at follow up. (Greene *et al* 2012, p.387-388, table 1).
Pickering *et al* 2013*	Student hand cleaning after toilet use<br>Student hand cleaning rate before lunch	"*Students at sanitizer intervention schools were over twofold more likely to clean their hands after toilet use than control school students (prevalence ratio = 2.2, 95% CI 1.2, 4.3), whereas students at soap intervention schools were not significantly more likely to clean their hands compared with students in control schools (prevalence ratio 1.0, 95% CI 0.3–3.8)*" (p.414)<br>"*Among all toileting events, the rate of hand cleaning with product (soap or sanitizer) was 82% at sanitizer schools (prevalence ratio 38.5, 95% CI 18.1–81.5), 37% at soap intervention schools (prevalence ratio 17.2, 95% CI 4.4–67.5), and 2% at control schools*" (p.414)<br>Mean proportion of students was not significantly different between schools: 0.90 at sanitizer schools (prevalence ratio 1.3, 95% CI 0.8–2.2), 0.82 at soap intervention schools (prevalence ratio 1.2, 95% CI 0.7–2.0), 0.69 at controls schools (p.414).

Study	Study outcome(s) presented	Results (effect of the intervention) and author conclusions
		"mean proportion of students cleaning hands with product before lunch was 0.61 at sanitizer schools (prevalence ratio 126.8, 95% CI 31.9–503.8), 0.70 at soap intervention schools (prevalence ratio 143.0, 95% CI 38.9–525.6), 0.01 at control schools" (p.415)
Rosen *et al.* 2006	Children washing hands with soap before lunch<br>Children washing hands with soap after bathroom use	Medium-term adjusted relative risk (RR) was 2.77 (CI: 1.70, 7.46, p < 0.01), long-term adjusted RR was 2.93 (CI 1.86, 6.97, p < 0.01). (p.30)<br>Medium-term adjusted RR was 2.90 (CI: 1.69, 10.06, p < 0.01), long-term adjusted RR = 3.30 (CI: 1.83, 16.67, p < 0.01) (p.30)<br>(Medium-term effect compares results 3 months after program launch in intervention with results before the end of the study period in the control. Long term effects compare results 6 months after program launch in intervention with results just before the end of study period in the control. Effect sizes were adjusted for religious sector and baseline handwashing levels, Rosen et al. 2006, p.28).
Stebbins *et al* 2011*	Behaviour change (students)	*"Students were observed to persist in meaningful and statistically significant improvements in their hand-washing frequency and in using hand sanitizer at least twice per day. The number of students using hand sanitizer four times per day significantly increased during flu season but did appear to drop off somewhat after flu season."* (p.318-20)<br>*"Students were observed to make and persist in meaningful and statistically significant improvements in covering coughs and sneezes, increasing their frequency of coughing into their elbow or shirt…. All responses were significantly higher in intervention than control schools"* (Stebbins *et al* 2010, p.320).
**Review outcome (h) Change in knowledge, attitudes or belief about hand hygiene**		
Freeman *et al* 2012*	Changes in pupil knowledge	*"We found significant and substantial differences in pupil knowledge between intervention and control groups after the intervention. Knowledge of key hand washing times and scores on a hand washing demonstration in intervention schools significantly increased."* (p.384, also table 2)<br>Mean number of students who mentioned two key hand washing times (before eating, after defecation): Intervention (1): 72 (SD=15) at baseline, 83 (SD=10) at follow up (p=0.09). Control: 75 (SD=14) at baseline, 78 (SD=12) at follow up. (table 2, p.385)
Pickering *et al* 2013*	Student perceptions of waterless hand sanitizer as an alternative to hand washing with soap and water<br>Teacher perceptions of waterless hand sanitizer as an alternative to hand washing with soap and water	*"91% of students at sanitizer schools stated that they would choose sanitizer to clean their hands over soap and water… they perceived cleaning hands with sanitizer to take a shorter time than hand washing with soap and water."* (p.415)<br>All teachers interviewed at sanitizer schools stated they would prefer provision of sanitizer over provision of soap at their school. (p.415)
Rosen *et al.* 2006	Pre-school educator beliefs<br>Pre-school educator attitudes<br>Pre-school educator knowledge	*"Beliefs about outcomes were positive toward hand washing in both groups (intervention: mean = 5.736, SD = 0.95; control: mean = 5.29, SD = 1.12). The effect of the intervention on beliefs about outcomes was borderline significant [least squares means (LSMeans) intervention 5.82, LSMeans control: 5.22, p = 0.0875, mixed models ANOVA]."* (p.692)<br>*"The effect of the interventions on attitudes was not significant (LSMeans intervention: 5.72, LSMeans group: 5.77, p = 0.9187, mixed models ANOVA)."* (p.692)<br>*"The score for the knowledge scale was 6.24 for the intervention group (SD = 0.73) and 5.81 for the control group (SD = 0.79). Knowledge was significantly higher in the intervention (LSMeans intervention group: 6.22, LSMeans control: 5.66, p = 0.0343"* (Rosen *et al* 2009, p.692)
Stebbins *et al* 2011*	Student knowledge	*"Intervention school students were observed to be more knowledgeable than control school counterparts."* (Stebbins *et* al. 2010, p.320 and table 4)
Uhari and Möttönen 1999	Knowledge of personnel	*"knowledge of infections… at the end of the trial was statistically significantly better at intervention centres in 3 of the 19 statements on the questionnaire, with no difference in the 16 other statements."* [*translated*]
